# Antimicrobial Fe_2_O_3_-CuO-P_2_O_5_ glasses

**DOI:** 10.1038/s41598-023-44743-x

**Published:** 2023-10-14

**Authors:** Alexandra L. Mitchell, Sung Hoon Lee, David J. McEnroe, Eric L. Null, Daniel A. Sternquist, Kathryn A. Hufziger, Brian J. Rice, Alex Scrimshire, Paul A. Bingham, Timothy M. Gross

**Affiliations:** 1 Corning Incorporated, 1 Riverfront Plaza, Corning, NY 14831 USA; 2 Corning Technology Center Korea, Corning Precision Materials Co., Ltd., 212 Tangjeong-ro, Asan, Chungcheongnam-do 31454 Republic of Korea; 3 Materials and Engineering Research Institute, Sheffield Hallam University, City Campus, Sheffield, S1 1WB UK

**Keywords:** Glasses, Biomedical materials

## Abstract

Glasses with high antimicrobial efficacy were developed in the Fe_2_O_3_-CuO-P_2_O_5_ ternary system to mitigate fomite-mediated transmission of infectious diseases in high-risk settings such as hospitals, daycares, and nursing homes. Binary CuO-P_2_O_5_ glasses were not durable enough for use as high touch point articles, so Fe_2_O_3_ was added to the compositions to increase the chemical durability. The amount of Cu leachate decreased by at least 3 orders of magnitude when Fe_2_O_3_ was increased from 0 to 13.1 mol%. At the highest Fe_2_O_3_ contents and corresponding highest durability, the glass was no longer able to pass a test of antimicrobial efficacy with < 3 log kill compared to > 5 log kill for all other compositions. *Ab-initio* molecular dynamics simulations showed increasing bridging oxygen species at the expense of non-bridging oxygen species with the increase in Fe_2_O_3_ content, showing that the glasses exhibited increased chemical durability because they were more interconnected and structurally bound. Experimental results with glasses at fixed CuO and decreasing Fe_2_O_3_ confirmed that Fe_2_O_3_ content (not CuO) controlled the Cu release rate and, thus, the antimicrobial efficacy of the glasses. The significance of the oxidation state of the leached Cu was overwhelmed by the importance of the amount of Cu leachate.

## Introduction

The COVID-19 pandemic emphasized that infectious disease outbreaks can have a significant effect on global health and the economy. While COVID-19 is primarily transmitted through respiratory droplets^1,2^, fomite-mediated transmission (through inanimate objects) is an important mode of transmission for many other infectious diseases such as methicillin-resistant *Staphylococcus aureus* (MRSA), vancomycin-resistant *Enterococcus *spp. (VRE), *Clostridium difficile*, *Acinetobacter *spp., *Pseudomonas aeruginosa*, norovirus, and rhinovirus^3–7^. Fomite transmission is of particular concern for nosocomial infections, as compliance with hand hygiene practices (the primary infection control method) is typically only 40–50% in health care settings^8^. Due to the significant risks of fomite-mediated transmission, there is a need for materials that possess antimicrobial (AM) and antiviral (AV) properties to reduce this transmission pathway.

In this study, black-colored, single-phase Fe_2_O_3_-CuO-P_2_O_5_ glasses (hereafter referred to as FeCuP glasses) were investigated. Optimized compositions exhibited comparable antimicrobial efficacy to Cu metal in a modified version of the Method for the Evaluation of Antimicrobial Activity of Hard, Nonporous Copper-Containing Surface Products^9^. The FeCuP glasses are phosphate glasses (Table 1). In contrast to many other phosphate glasses, they can be melted and delivered under normal atmospheric conditions. However, a thin (~ 2–3 micron) layer of Cu and O-rich surface crystallization formed upon cooling in an oxygenated atmosphere. To prevent this surface crystallization, all the glasses discussed in this paper were delivered in an inert, Ar atmosphere. The glasses can be cast or machined into bulk parts and are primarily being investigated for use as high touch point articles (e.g., elevator buttons, door push plates) in architectural applications such as healthcare and office settings.

**Table 1:** FeCuP glass compositions and properties (Series A = Glasses #1–#4 and Series B = Glasses #4–#6).

Renormalized, analyzed composition (mol%)	﻿1	2	3	4	5	6
P_2_O_5_	46.6	46.2	45.7	45.1	48.1	50.0
Fe_2_O_3_	0.0	4.3	8.7	13.2	9.2	8.9
CuO	53.4	49.5	45.6	41.6	42.7	41.0
Log kill	> 5	> 5	> 5	1.8 ± 0.3	> 5	> 5
Fe^2+^/total Fe	–	0.42 ± 0.02	0.47 ± 0.02	0.65 ± 0.02	0.60 ± 0.02	0.55 ± 0.02
Cu^1+^/total Cu	0.49	0.48	0.72	0.87	0.57	0.57
Total reducing power	0.49	0.48	0.65	0.78	0.58	0.57
mol Cu^1+^	0.240	0.214	0.284	0.304	0.208	0.198
Total mol Cu	0.491	0.442	0.395	0.350	0.363	0.346
Crystalline species	None	None	None	Cu_4_(PO_4_)_2_O CuFeO_2_	None	None
Color	Black	Black	Black	Black	Black	Black

The most important property of these glasses is chemical durability. Compositions with insufficient durability reacted with ambient moisture and dissolved, resulting in poor reliability for use as high touch point articles. If the glasses were too durable, then Cu could not leach from the surface, resulting in poor antimicrobial efficacy. This study varied the chemical durability of the glasses by changing the glass composition (and, consequently, the glass structure). Using *ab-initio* molecular dynamics (AIMD) simulations and leaching experiments, it was found that increasing Fe_2_O_3_ contents increased the glass polymerization such that above a certain threshold, not enough Cu was released to exhibit high AM efficacy. Cu redox was also found to contribute to AM efficacy, though the oxidation state of the Cu was not as important as the total amount of Cu released from the surface of the glass. In this study, six glasses were investigated to understand the AM behavior in the FeCuP system as a function of composition. These compositions fall within one of the very few areas of composition space that have exhibited > 5 log reduction in antimicrobial testing, so identifying the mechanism driving the AM behavior will help with composition optimization as well as with future AM material design.

## Results

**Figure 1:**
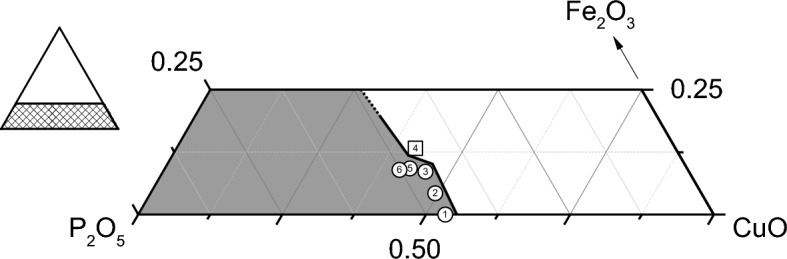
Ternary diagram (0.25Fe_2_O_3_–CuO–P_2_O_5_) showing the glass forming region in grey and the six glass compositions of interest.

Six glass compositions were investigated (Table 1, Fig. 1). In the first four compositions (Series A), the Fe_2_O_3_ content increased in increments of 5 mol% at the expense of CuO while batched P_2_O_5_ was held constant at 45 mol%. In the fourth, fifth, and sixth compositions (Series B), batched CuO was held constant at 40 mol%, and batched Fe_2_O_3_ decreased by 2.5 mol% with increasing P_2_O_5_. The analyzed compositions (Table 1) varied slightly from the batched targets due in part to reaction with the quartz crucibles, resulting in tramp SiO_2_. Compositions shown in Table 1 and in all figures were renormalized to exclude the tramp SiO_2_ (0.3–1.3 mol%). The AIMD simulations were based on the renormalized, analyzed compositions coupled with the measured oxidation state data. Values used for the AIMD simulations are reported in Supplementary Table 1. Full, analyzed compositions (with SiO_2_) are included in Supplementary Table 2.

All glasses except glass #4 were fully amorphous, as determined by XRD. Glass #4 contained minor amounts of crystalline Cu_4_(PO_4_)_2_O and CuFeO_2_ (Supplementary Fig. 1). Cu fluorescence elevated the background signal, and the XRD peaks were too low above the background for the phase proportions to be determined by Rietveld refinement. Further, when glass #4 was examined via SEM, no crystalline phases were observed, indicating that the crystallinity was minor and likely did not have a significant effect on the leaching or AM efficacy data discussed below.

### Antimicrobial efficacy

The glasses discussed here (apart from the minor crystallinity in glass #4) are single phase amorphous materials with high Cu contents. The primary mechanism of antimicrobial efficacy is the presence and release of Cu ions from the glass surface, where the effectiveness of the Cu decreases with increasing oxidation state (Cu^0^ > Cu^1+^ > Cu^2+^)^10^. Five of the six glasses exhibited high antimicrobial efficacy, with > 5 log kill of the *Staphylococcus aureus* bacteria that were spread onto the glass coupon surface and incubated for 120 min under ambient laboratory conditions (see “Methods” section for full details). The remaining glass composition (#4) exhibited 1.8 ± 0.3 log kill, below the critical ≥ 3 log kill threshold for AM efficacy (Table 1). Low AM efficacy can be caused by low amounts of Cu release from the glass surface (high glass chemical durability), Cu that is too oxidized to effectively kill bacteria, or a combination of both. For the six glasses investigated here, the Cu leach rate and Cu redox were measured to determine chemical durability and oxidation state as a function of glass composition. *Ab-initio* molecular dynamics simulations were then used to determine the glass structure as a function of composition to ascertain why some glasses in this system exhibited > 5 log kill and others did not.

### Chemical durability

**Figure 2:**
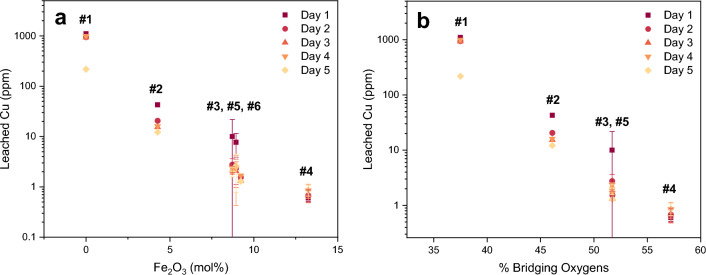
(**a**) The amount of leached Cu decreases on a log scale as a function of increasing Fe_2_O_3_ content, emphasizing the increased aqueous chemical durability with Fe_2_O_3_ content. Glass #s are labelled above each composition. Data plotted is renormalized to exclude SiO_2_. (**b**) Leached Cu also decreases as a function of % bridging oxygens, showing that as the glass structure became more interconnected (more bridging oxygens), less Cu could be released from the surface. Note that bridging oxygens are defined as oxygens that link neighboring P- and Fe-polyhedra. These values were calculated from the AIMD simulation results. Glass #6 is not shown as the modeled composition was modified to contain significantly less Fe_2_O_3_ than the experimentally obtained glass.

Chemical durability was evaluated by leaching FeCuP glass coupons in DI water and measuring the amount of the leached components (Fe, Cu, and P) after each day, where the DI water was sampled and replaced every day for five days. With increasing Fe_2_O_3_ in the glass, the amount of leached Cu decreased linearly on a log scale (Fig. 2a; all six compositions). Thus, higher Fe_2_O_3_ content compositions exhibited significantly higher chemical durability than compositions with low or no Fe_2_O_3_. Leached Cu also decreased linearly on a log scale as a function of total bridging oxygens (as determined by the AIMD simulations; shown in Fig. 2b), indicating lower dissolution rates with an increasingly interconnected glass structure (more bridging oxygens).

**Figure 3:**
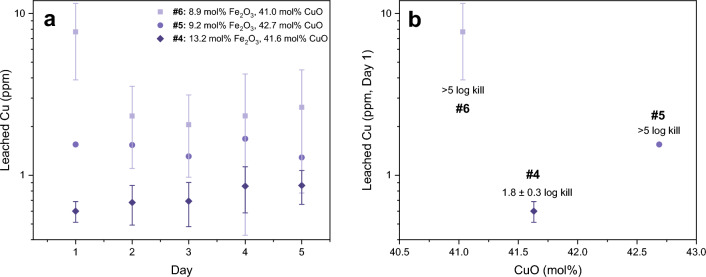
(**a**) Glasses with higher Fe_2_O_3_ contents leached less Cu on each sampling day, indicating greater aqueous chemical durability. More Cu was leached at lower Fe_2_O_3_ contents even if there was less total CuO in the composition (e.g., light purple squares with 41.0 mol% CuO compared to dark purple diamonds with 41.6 mol% CuO). (**b**) Glass #4 (1.8 ± 0.3 log kill) showed low AM efficacy (< 3 log kill) despite having a comparable or greater amount of total CuO than glasses #5 and #6. Day 1 data is shown as the most comparable leach data to the conditions of the modified U.S. E.P.A. test. Glass #s are labelled above each composition. Data plotted is renormalized to exclude SiO_2_.

At comparable total CuO in Series B (glasses #4–#6), the glass compositions with the highest Fe_2_O_3_ leached the least Cu on days 1, 2, and 3 (Fig. 3a). On days 4 and 5, the difference in the amount of leached Cu was indistinguishable within uncertainty for all three Series B glasses. The amount of Cu leached from the surface after days 1, 2, and 3 was dependent on the Fe_2_O_3_ content and independent of the total amount of CuO in the bulk composition.

Figure 3b shows the day 1 leached Cu as a function of Cu content (mol%), the conditions during the leaching experiment that were expected to be most like the conditions in the AM efficacy test (compared to the leach data obtained on days 2, 3, 4, or 5). The glass with the least amount of leached Cu (glass #4, dark purple diamond) was also the only glass that did not perform well in the AM efficacy test. This composition had comparable CuO contents to both glasses #5 and #6, where it contained 0.7 mol% more CuO than glass #6 (Fig. 3b). These results show that the low amount of leached Cu observed from glass #4 was not caused by low total CuO contents in the glass. In the discussion, it is proposed that low Cu leach rates caused glass #4 to exhibit low AM efficacy due to a more structurally bound (highly durable) composition.

When the ratios of the leached atoms were compared to the initial atomic ratios in the glasses, it was shown that the glasses dissolved incongruently (Supplementary Table 3). When normalized to Cu (as the largest component of the leachate) and compared to the initial atomic ratios in the glass, about 46% of the expected P was released and about 80% of the expected Fe was released for the day 5 leachate data. Such incongruent dissolution suggests that a Cu depleted layer could eventually form on the glass surface, though longer duration and more rigorous testing relevant to the specific end use/product format is needed to fully characterize and quantify such an effect. This will be addressed in a future publication.

### Cu and Fe redox

The oxidation state of the released Cu is significant in determining how effective a material can be at killing bacteria and viruses. Glass redox can be significantly affected by melting and processing conditions. In this study, melting and processing parameters were standardized to determine the effect of composition on the oxidation state of the glass.

**Figure 4:**
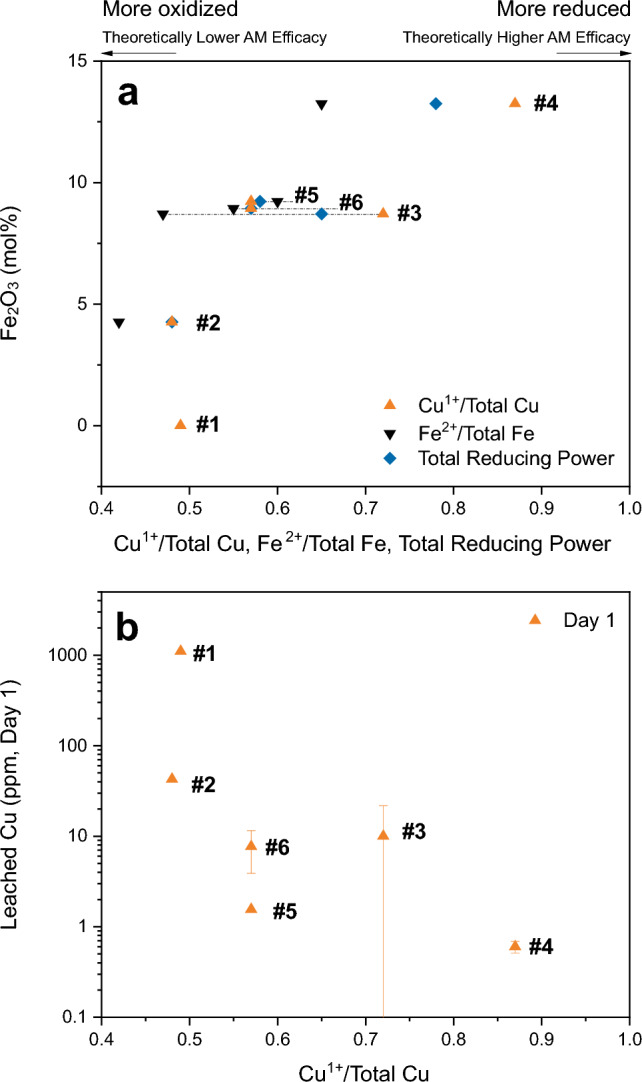
(**a**) Fe_2_O_3_ content (mol%) v. Cu^1+^/Total Cu, Fe^2+^/Total Fe, and total reducing power $\left( {\left[ {{\text{Cu}}^{1 + } } \right] + \left[ {{\text{Fe}}^{2 + } } \right]} \right)/\left( {\left[ {{\text{Cu}}} \right] + \left[ {{\text{Fe}}} \right]} \right)$ showing that the highest Fe_2_O_3_ content glass (#4) was the most reduced. Despite being the most reduced, glass #4 was the only composition that did not have high AM efficacy. Glass #s are labelled next to the three values for each composition. Horizontal dashed lines are used to distinguish the data for glasses #3, #5, and #6 as they have similar Fe_2_O_3_ contents. Data plotted is renormalized to exclude SiO_2_. Cu^1+^/Total Cu is only 0.01 greater than the total reducing power for glass #2 because the amount of Fe is just 13% of the total moles of redox species. (**b**) Leached Cu (ppm) after Day 1 on a log scale as a function of Cu^1+^/Total Cu. The glass with the most Fe_2_O_3_ contained the most reduced Cu, which should have had the highest AM efficacy. However, high AM efficacy was not observed with this composition likely due to the low amount of total Cu released, as shown in (**b**).

Total reducing power was measured using a combination of Cr/Cu and Cr/Fe redox reactions (Table 1; see “Methods”). Glasses #2–#6 contained two multivalent species, Cu and Fe. For these compositions, the Fe^2+/3+^ ratio was estimated using Mössbauer spectroscopy (Supplementary Table 4 and Supplementary Fig. 2), then the Cu^1+/2+^ ratio was calculated for each composition using the measured total reducing power values and approximate Fe^2+/3+^ ratios (Table 1, Fig. 4; see “Methods” section for details). For the Series A glasses with both multivalent species (#2-#4), as the Fe_2_O_3_ content of the glass increased from 4.3 to 13.2 mol%, the total reducing power increased and both the Fe and the Cu became more reduced (Fig. 4a). Then, in the Series B glasses (#4–#6), as the Fe_2_O_3_ content of the glass decreased from 13.2 to 8.9 mol%, the total reducing power decreased and both the Fe and Cu became more oxidized (Fig. 4a). Of all six glasses, glass #4, the composition with the highest Fe_2_O_3_ content and the lowest AM efficacy, contained the most reduced Cu.

Cu is most effective for AM efficacy in its most reduced state (Cu metal) followed by Cu^1+^ and then Cu^2+^^10^. Thus, compositions with more reduced Cu should be more effective in terms of AM efficacy per Cu ion released from the surface. The glass with the most reduced Cu in this study (glass #4), however, was the lone composition that showed low AM efficacy. As mentioned above, this composition leached the least amount of Cu (Fig. 4b), so while the Cu species that were released should have had higher antimicrobial efficacy, not enough Cu was released to kill > 99.9% of the *Staphylococcus aureus.*

### Glass structure

*Ab-initio* molecular dynamics simulations were performed using modified versions of the renormalized, analyzed glass compositions which were corrected to account for the oxidation states of the Cu and Fe. For glasses #2, #3, #5, and #6, too many atoms were required to model the system containing P_2_O_5_, Cu_2_O, CuO, FeO, and Fe_2_O_3_ (497, 503, 500, and 1016 atoms, respectively). To simplify those compositions, all Fe was accounted for as FeO instead of as FeO and Fe_2_O_3_ as there was < 3 mol% Fe_2_O_3_ in each of the glasses. Glass #4 was used to test the hypothesis that treating all Fe as FeO would not change the glass structures within the uncertainty of the simulation results. For all bond types present at fractions larger than 1.3% (P–O–x, P–O–Fe, and P–O–P), there was a difference of < 3.2% between the case where the simulations were run with all five oxide components compared to when they were run with only four oxide components (i.e., all Fe as FeO; Supplementary Table 5). For the three bond types that were present in minor amounts (Fe–O–x, Fe–O–Fe, and P–O–Fe, Fe), there was a much larger percent difference between the two cases (89%, 65%, and 27%, respectively). The absolute difference in these three cases, however, was only 0.2%, 1.2% and 0.3%. The observed changes were within the errors of the simulations, suggesting that a simplified four component system for glasses #2, #3, #5, and #6 could be used to determine the changes in the glass structure with increasing Fe_2_O_3_ content at the expense of CuO (Series A) and decreasing Fe_2_O_3_ at the expense of P_2_O_5_ (Series B).

In addition to the simplification of the compositions, the simulated glass #6 was modified from the experimentally analyzed composition because the compositions for glasses #5 and #6 would have been indistinguishable within the error of the simulations. The modified glass #6 maintained constant CuO and contained less Fe_2_O_3_ and more P_2_O_5_ than was obtained in the experiments (Supplementary Table 1). Because the composition was modified, glass #6 is omitted from Fig. 2b, which shows experimentally measured values as a function of the simulated % bridging oxygens.

**Figure 5:**
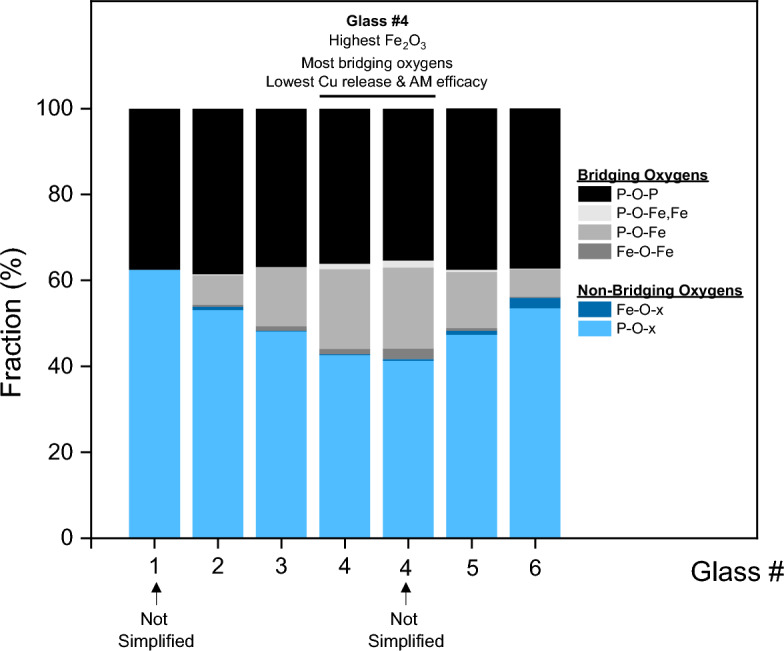
*Ab-initio* molecular dynamics simulations based on analyzed compositions. Glass #1 was not simplified, and glasses #2–#6 were simplified to obtain reasonable computation times. Glass #4 was also run without simplification to assess the validity of the simplification assumption (shown as the second #4 column). The AIMD results showed a more connected glass network at higher Fe_2_O_3_ contents via an increasing fraction of bridging oxygen species (P–O–P, Fe–O–Fe, and P–O–Fe) with a corresponding decreasing fraction of non-bridging oxygen species (P–O–x and Fe–O–x) as a function of Fe_2_O_3_ content for Series A glasses with fixed P_2_O_5_ at 45 mol% and (55-X_Fe2O3_) mol% CuO and for Series B glasses with fixed CuO at 42 mol% and (58-X_Fe2O3_) mol% P_2_O_5_.

Figure 5 shows the AIMD results, where glasses #2-#6 are shown using the simplified four component system for consistent comparison. Also shown in Fig. 5 are glass #1, which does not contain Fe and did not require simplification, and the more accurate five component representation of glass #4 for comparison with the simplified case. Structural properties such as densities, coordination numbers (CN) of the elements, and radial distribution functions (RDF) were calculated to determine the changes in the glass structure with composition. For each composition, 5 independent structures were generated using a simulated melt and quench process (see “Methods”). Radial distribution functions were indistinguishable for P–O, Cu–O, Fe–O, P–P, Cu–Cu and Fe–Fe. It was also found that the average coordination number for oxygen increased for Series A glasses, then decreased for Series B glasses (Supplementary Fig. 3), such that the highest average coordination number for oxygen was found in the glass with the highest Fe_2_O_3_ content. The ratio of the bridging oxygens to non-bridging oxygens increased significantly with increasing Fe_2_O_3_ content for both series such that glass #4, the composition with the most Fe_2_O_3_, had the highest value (Fig. 5). With increasing Fe_2_O_3_ in both series (Series A: glass #1→#4 and Series B: glass #6→#4), more and more P–O–Fe, Fe–O–Fe, and P–O–(Fe, Fe) bridging oxygen bonds formed at the expense of the non-bridging P–O–x bonds (Supplementary Table 5). Fe–O–x bonds also decreased slightly, though not as significantly as the P–O–x bonds. The increase in bridging oxygens indicated that the glasses were becoming more structurally bound and interconnected with increasing Fe_2_O_3_ content. With the addition of Fe_2_O_3_, increased polyhedron edge sharing was observed with a peak of ~ 5% in glass #4 (13.2 mol% Fe_2_O_3_). The edge sharing comes from FeO_6_–FeO_6_ polyhedron and was observed at the expense of corner sharing, which was 100% in the glass that did not contain Fe_2_O_3_. Overall, the introduction of Fe_2_O_3_ and the increase in Fe_2_O_3_ in the Series A and Series B glasses resulted in the creation of FeO_6_ octahedra and an increase in bridging oxygens.

## Discussion

### Chemical durability

While copper phosphate glasses exhibit high AM efficacy, they react with ambient moisture and dissolve forming a Cu-infused, phosphoric acid gel. Such surface reaction and dissolution is not practical for high touch-point articles, so Fe_2_O_3_ was used to increase the glass’ chemical durability. Fe_2_O_3_ is known to increase the chemical durability of phosphate glasses and has long been used in glasses designed for high-level, radioactive nuclear waste encapsulation^11,12^.

The compositions in Series A were chosen to identify the Fe_2_O_3_ threshold above which there would no longer be sufficient Cu release from the surface for the glasses to exhibit high AM efficacy (where high AM efficacy is defined as log kill ≥ 3). At elevated Fe_2_O_3_ content (e.g., 13.1 mol% in glass #4), the glass became so durable that Cu could not be easily leached from the surface, and the glass exhibited low AM efficacy. Because the increase in Fe_2_O_3_ in Series A was accompanied by a decrease in CuO (the antimicrobial component), Series B was designed to test whether high AM efficacy could be achieved at the lowest CuO content by reducing the Fe_2_O_3_ content in the glass. In both glasses #5 and #6, the durability decreased with the decrease in Fe_2_O_3,_ and the glasses exhibited high AM efficacy despite containing the lowest total CuO. Combined, these two series of glasses showed that the chemical durability of the glasses in the FeCuP system can be controlled by the Fe_2_O_3_ content of the glass, such that the log of the leached Cu, P, and Fe decreased linearly with increasing Fe_2_O_3_ (Fig. 2, Supplementary Fig. 4). It was also shown that at ~ 40 mol% CuO, it was not the glass’ Cu content that prevented glass #4 from exhibiting high AM efficacy.

The relationship between glass composition and chemical durability was further observed in the results from the molecular dynamics simulations. In the Series A glasses, the glass structure became increasingly interconnected and polymerized with increasing Fe_2_O_3_ content, as evidenced by the increase in bridging oxygen species (Fig. 5) and the corresponding decrease in non-bridging oxygens. Most phosphate glasses exhibit low chemical durability due to phosphate chain hydration. In this process, protonation occurs on both terminal phosphate groups of each phosphate chain that is dissolved from the glass^13^. The chains are dissolved as units, and phosphate groups in the middle of the chains are not protonated. Dissolution of phosphate glasses through cleavage of P–O–P bonds by hydrolysis was not found to cause high dissolution rates as the rates of chain hydrolysis are an order of magnitude slower than the observed dissolution rates^13^.

For the glasses discussed here, the Fe_2_O_3_ caused the glass network to become more polymerized by increasing the number of bridging oxygens through an increase in hydration-resistant P–O–(Fe, Fe), P–O–Fe, and Fe–O–Fe bonds. This is supported by the AIMD simulations and the fitted data from Mössbauer spectroscopy (Fig. 5, Supplementary Table 4, and Supplementary Fig. 2). The fitted Center Shift (CS) and Quadrupole Splitting (QS) values are consistent with both the Fe^2+^ and the Fe^3+^ ions occupying a range of distorted octahedral sites^14–16^. The increasing polymerization with Fe_2_O_3_ content led to an increase in the chemical durability (Fig. 2b). Several hypotheses to explain why iron increases the chemical durability of phosphate glasses have been reported in the literature, including that iron could (1) potentially strengthen the cross-bonding between the polyphosphate chains^13,17^, (2) strengthen the bonding of the ends of the chains to the surrounding glass^12^, and/or (3) replace P–O–P bonds with more hydration-resistant P–O–Fe bonds^18^. In this study, most of the hydration-resistant bonds formed at the expense of the non-bridging oxygen P–O–x bonds, not the P–O–P bonds (Fig. 5), suggesting that the third hypothesis was not relevant in this system. The creation of the P–O–(Fe, Fe), P–O–Fe, and Fe–O–Fe bonds at the expense of the non-bridging oxygen bonds supports the first and second hypotheses that (1) Fe was cross-bonding or cross-linking the phosphate chains in the FeCuP system like metal chelate structures, as proposed by Van Wazer and Campanella^17^ and (2) that Fe was strengthening the bonding at the end of the chains by reducing the number of non-bridging oxygens. This behavior is also consistent with similar structural behavior observed by a wide body of research into transition metal phosphate glasses, with potential applications ranging from biomedical to nuclear waste immobilization^14–16,19^. The experimental and modeling results suggest that the increase in chemical durability was caused by a more interconnected and bound glass structure at higher Fe_2_O_3_ contents. Between 9.1 and 13.1 mol% Fe_2_O_3_, there was a threshold above which the FeCuP glass structure was too structurally bound to release enough Cu to exhibit high AM efficacy.

### Cu redox

In addition to the total amount of Cu released from the surface, the oxidation state of the released Cu is important for determining a material’s AM efficacy. Cu is most effective in its most reduced state (Cu metal, Cu^0^), then becomes less effective at killing bacteria and viruses with increasing oxidation state (Cu^0^ > Cu^1+^ > Cu^2+^)^10^. Cu^1+^/Total Cu was determined for all six glasses. Glass #4, the only composition that showed low AM efficacy, had the highest Cu^1+^/Total Cu ratio and the most moles of Cu^1+^ (Table 1, Fig. 4b)_._ As the most reduced glass in the series, the AM efficacy of the Cu released from the surface of glass #4 was expected to be the highest of the four glasses in Series A. However, even with the most antimicrobial Cu species, not enough Cu was released to achieve high AM efficacy (Fig. 2a). As discussed above, the glasses in Series B showed that the high Fe_2_O_3_ contents (not the low CuO contents) in glass #4 caused the increased polymerization of the network, resulting in the low leach rates. So, while glass #4 had the lowest total CuO content in Series A, it was the high Fe_2_O_3_ content that prevented the glass from achieving high AM efficacy. These conclusions highlight that although Cu redox was a contributing factor to the AM efficacy of the FeCuP glasses, the contribution of Cu redox to AM efficacy was overwhelmed by the amount of released Cu. This was seen in glasses #1, #2, #3, #5, and #6, which contained more oxidized Cu but released more of it (Table 1).

### Antimicrobial efficacy

Copper has been used for medicinal purposes such as sterilizing chest wounds and drinking water since at least 2600–2200 B.C., when it was documented in the Egyptian medical text the *Smith Papyrus*^20^. Since that time, Cu has been used in civilizations around the world for antimicrobial purposes, though usage decreased with the advent of antibiotics in the 1930s. With the rise of antimicrobial resistance, particularly in hospital settings, Cu and Cu-containing materials have once again gained interest as a way to reduce fomite transmission of nosocomial infections through contact killing of bacteria and viruses.

Though several mechanisms for Cu contact killing have been described, there is no universally accepted model^21^. It is possible that the observed mechanisms work in concert with one another in some cases but in isolation for others. The bacteria/virus of interest as well as the contact surface are important in determining the dominant mechanism for the AM behavior of Cu.

The first mechanism that is often described is cell membrane damage and subsequent cell death caused by Cu ions released from the matrix of the contact material. For *Staphylococcus haemolyticus*, for example, it was shown that cell death was caused by membrane damage^22^. In that study, no more DNA mutation was observed than was found in bacteria exposed to stainless steel. Similar evidence for loss of membrane integrity was found for *E. coli*^23^. The second mechanism is oxidative stress caused by the creation of reactive oxidative species (ROS) from redox cycling between the three oxidation states of Cu^20,24^. The third mechanism is DNA degradation, where some studies have observed cell membrane deterioration followed by DNA degradation^24,25^. Regardless of which of these mechanisms (or which combination of them) is occurring in the FeCuP glasses, many of these studies have found that the amount of Cu release is critical for determining AM efficacy, similar to the findings presented here^20,21,26–28^.

## Conclusion

This paper discussed black, antimicrobial glasses in the Fe_2_O_3_-CuO-P_2_O_5_ system for the first time. Chemical durability was the most critical glass property as it determined both the amount of Cu release (the most critical parameter for AM efficacy in this system) as well as whether the glasses would react and dissolve under ambient conditions. To increase the chemical durability, Fe_2_O_3_ was added to the glasses. Higher Fe_2_O_3_ contents resulted in higher chemical durability such that the highest Fe_2_O_3_ content glass was so durable that not enough Cu was released to achieve ≥ 3 log kill in the test of antimicrobial efficacy. It was found that Fe_2_O_3_ increased the bridging oxygen species in these glasses at the expense of the non-bridging oxygen species. This increased the polymerization of the glasses and prevented dissolution. Cu redox was not as important a parameter as the total amount of leached Cu for AM efficacy, where the Fe_2_O_3_ content determined the total amount of Cu release for the glasses in this study. In summary, FeCuP glasses were described, and the relationship between glass composition, glass structure, and antimicrobial efficacy was explored.

## Methods

### Glass preparation

Glasses were prepared using ammonium dihydrogen phosphate (NH_4_H_2_PO_4_; BassTech), iron (III) phosphate (FePO_4_⋅2H_2_O; Alfa Aesar), and cupric oxide (CuO; American Chemet; 325 mesh). Raw materials were mixed in a Turbula^®^ mixer for 45 min, then calcined overnight at 250 °C in a 650-cc fused quartz crucible with a refractory silica lid. Glasses were melted at 850 °C for 1 h, then 1100 °C for 30 min in the fused quartz crucible with the lid. Glasses were then poured into graphite molds that were pre-heated to 350 °C. The top, exposed surfaces of the glasses were immediately placed in an inert Ar atmosphere. The Ar flowed above the top of each glass and another heated mold was placed on top to create a sealed environment. Once the surface of the glass was quenched, the Ar gas was turned off, and the glass was allowed to slow cool in the graphite molds. The resulting cast disks were 152.4 mm in diameter, ~ 12.7 mm thick, and had a glossy black appearance on the top surface. The inert atmosphere was critical for preventing a thin layer of surface crystallization from reaction with oxygen. The glasses had very low annealing temperatures, so slow cooling the glasses in the molds relieved thermal stresses, and a subsequent annealing step was not required.

Glass coupons were prepared by cutting 25 mm × 25 mm × 5 mm parts from the glass disks. These were ground and polished on both flat surfaces down to 3 mm thickness using an aqueous solution. The parts were cleaned using isopropyl alcohol and a Kimwipe^®^. Then, glass coupons were polished further using a film of 0.3-micron grit alumina with isopropyl alcohol. This step exposed a new surface that did not contact an aqueous solution, creating a fresh surface for the leaching experiments and AM efficacy testing. After sample preparation, but prior to experimentation and testing, samples were stored in a glove box to prevent surface reactions with oxygen and atmospheric moisture.

### Chemistry

The chemical compositions of the glasses were measured using inductively coupled plasma-optical emission spectroscopy (ICP-OES). A 0.05 g portion of each sample was weighed and placed in a Digitube bottle, followed by 5 mL of deionized (DI) water, 4 mL of 1:1 nitric acid (HNO_3_), 4 mL of 1:1 hydrochloric acid (HCl), and 1 mL of hydrofluoric acid (HF). The solution was then placed in a hot block until the digestion was completed (~ 2–8 h). After digestion, the solution was transferred to a 500 mL flask containing 10 mL of nitric and hydrochloric acids such that the final acid concentrations were approximately 2% HNO_3_, 2% HCl, and 0.2% HF. Each sample solution was then analyzed using a Perkin Elmer Optima 7300V Simultaneous ICP-OES with a matrix-matched standard and blank to determine the concentrations of Fe, Cu, P, and Si.

### X-ray diffraction

Glass samples were ground to a fine powder in a Rocklabs ring mill. The powders were then pressed into a stainless steel back-fill sample holder, loaded into a Bruker D4 or D8 Endeavor equipped with Cu radiation and a Lynx Eye detector, and scanned from 5 to 80° 2θ for a total time of 12 min. The resulting data was analyzed in MDI Jade. The crystalline phase(s) were identified using a combination of the Jade search match and the ICDD’s PDF-4 manual search.

### Antimicrobial efficacy

Bactericidal efficacy tests were conducted using a modified version of the U.S. E.P.A. Method for the Evaluation of Antimicrobial Activity of Hard, Nonporous Copper-Containing Surface Products^9^. Each glass coupon was tested in duplicate. A 20-µL aliquot of thawed *Staphylococcus aureus* bacterial cultures was added to 10 mL Tryptic Soy Broth (Teknova). These bacterial suspensions were incubated at 36 °C for 18–24 h. The suspension was then centrifuged at 5000 rpm for 10 min, after which the supernatant was removed, and the pellet was re-suspended in 5 mL of 1X phosphate buffered saline. An organic soil load containing 0.25 mL of 5% fetal bovine serum (Gibco Life Technologies) and 0.05 mL Triton X-100 (Amresco Pro Pure) was added to 4.70 mL bacterial suspension to aid in spreading the inoculum. The optical density of the inoculum was recorded.

Each coupon was inoculated with 20 µL of the bacterial test culture. The inoculum volume was spread evenly using bent sterile pipette tips (Mettler-Toledo) to ensure full and even coverage, spreading as close to the edge of the coupon as possible. Coupons were then incubated under ambient laboratory conditions for a period of 120 min. Following the 120 min exposure period, coupons were neutralized in 20 mL Letheen broth (Hardy Diagnostics). Ten-fold serial dilutions of the neutralized solutions were plated using standard spread plate technique on Tryptic Soy Agar plates and incubated for 24 or 48 h at 36 °C to yield countable numbers of survivors (approximately 20–200 colonies per plate).

### Leaching study

Leach testing was conducted on two separate coupons for glasses #3, #4, and #6 and on a single coupon tested for other compositions. The limited number of test coupons available was due to the desire to conduct all experiments and measurements on glass from the same melt pour to keep the redox state of the Cu and Fe in the glass consistent across measurements and tests. Each coupon was submerged in 15 mL DI water in a covered container and leached for a total of 5 days at ambient temperature. On days 1, 2, 3, 4, and 5, the entire 15 mL volume was removed for analysis, the coupon briefly rinsed with deionized water, and again submerged in 15 mL of fresh DI water. When multiple coupons were tested, the leachate was analyzed separately rather than combined into a single aliquot, and error bars are shown as the standard deviation of the two measurements. Testing was not extended past five days due to the significant decrease in the leached elements. Leachate was analyzed using an Agilent 7700s ICP-MS with results reported for Fe, Cu, and P.

### Total reducing power, Fe redox (Mössbauer spectroscopy), and Cu redox

The glasses in this study contained the following multivalent species: only Cu or Cu and Fe. The redox states of the Cu and Fe were measured using a combination of inductively coupled plasma–optical emission spectroscopy (ICP–OES) to determine the total Cu and Fe contents and Cr/Cu and Cr/Fe redox reactions to determine total reducing power of the glass. Mössbauer spectroscopy was used to measure the Fe^2+^/total Fe ratio.

To determine the total reducing power of glasses #1-#4, 0.5 g of crushed sample was dissolved with diluted sulfuric and hydrofluoric acids (5 mL H_2_SO_4_, 5 mL HF, and ~ 90 mL H_2_O). To determine the total reducing power of glasses #5 and #6, 0.05 g of ground sample was dissolved in the same diluted acids. For all the glasses, a known amount of Cr^6+^ was added to the solutions in the form of K_2_Cr_2_O_7_. The amounts added were expected to oxidize any reduced species and provide a small excess. Upon the addition of the Cr^6+^, the following two reactions occurred if both Cu and Fe were present, and only reaction (1) occurred if there was no Fe_2_O_3_ in the glass (Eqs. 1, 2):

1 $${\text{3 Cu}}^{{{1} + }} + {\text{ Cr}}^{{{6} + }} \to {\text{3 Cu}}^{{{2} + }} + {\text{ Cr}}^{{{3} + }}$$

2 $${\text{3 Fe}}^{{{2} + }} + {\text{ Cr}}^{{{6} + }} \to {\text{3 Fe}}^{{{3} + }} + {\text{ Cr}}^{{{3} + }}$$

The amount of excess Cr^6+^ was measured by a titration with Fe^2+^. For glass #1, the Cu^1+^/Total Cu ratio was determined. For glasses #2–#6, the equivalence total of Fe^2+^ and Cu^1+^ was measured. The total reducing power reported is the total moles of reduced species divided by the total moles of multivalent species (Eq. 3).

3 $$\left( {\left[ {{\text{Cu}}^{1 + } } \right] + \left[ {{\text{Fe}}^{2 + } } \right]} \right)/\left( {\left[ {{\text{Cu}}} \right] + \left[ {{\text{Fe}}} \right]} \right)$$

The total measured reducing power (numerator) was determined using the redox reaction and titration described above (in milliequivalent/gram units), and the total available reducing power of multivalent species (denominator) was determined using the measured ICP–OES data. The total available reducing power assumes that all the Fe and Cu are present in their reduced forms. Total reducing power can vary between 0 and 1, where 0 means that there are no reduced species present, and 1 means that there are no oxidized species present.

For glasses #2–#6, Mössbauer spectroscopy was used to determine the Fe^2+^/Total Fe ratio, then the Cu^1+^/Total Cu was calculated.

^57^Fe Mössbauer spectroscopy measurements utilized acrylic absorber discs with a sample area of 1.767 cm^2^. The disks were loaded to present 2.16 × 10^−3^ g cm^−2^ of Fe and achieve a Mössbauer thickness of 1^29,30^. Samples were homogeneously mixed with graphite to achieve this level of loading. The 14.4 keV γ-rays were supplied by the cascade decay of 25 mCi ^57^Co in a Rh matrix source and were oscillated at constant acceleration. All measurements were calibrated relative to α-Fe foil. Spectral data were fitted using the Recoil software package^31^ using a Voigt-based fitting approach^32^. The fitted areas for individual doublet components enabled an estimation of the Fe^2+^/Total Fe ratio. However, as noted in previous publications, the recoil-free fraction ratio, *f*(Fe^3+^)/*f*(Fe^2+^), which is often assumed to have a value of 1 for oxide glasses, is 1.3 for other phosphate glasses which are compositionally very broadly similar to the glasses studied here^14–16^. Consequently, the estimations of redox ratios here assume that *f*(Fe^3+^)/*f*(Fe^2+^) = 1.3 (Supplementary Table 4).

### Ab-initio molecular dynamics simulations

To generate glass structures of each composition, *ab-initio* molecular dynamics (AIMD) was adopted. Initially, the atomic elements for a specific composition were randomly distributed in a fixed volume box and heated to a temperature of 4000 K for 5 ps (NVT) to mimic experimental melting process. The system was then subjected to a quenching cycle to rapidly cool it down to 300 K for 30 ps. At 300 K, the system was equilibrated for additional 2.5 ps, followed by a final optimization step of constant energy (NVE) run for 2.5 ps to achieve an equilibrium atomic bonding arrangement. To ensure the accuracy of the results, 5 structures were generated for a given composition and were analyzed to obtain a static set of properties. This methodology has been successfully employed in previous studies to predict both structural and electronic properties. More detailed methodology can be found in a previous report^33^.

AIMD calculations were performed with the *Vienna ab-initio simulation package* (VASP)^34,35^, and the generalized gradient approximation (GGA) was used for the exchange–correlation functional^36^. A cutoff energy was set as 430 eV with single point k-mesh, and core electrons were incorporated with pseudopotentials with the projector augmented wave (PAW) method^37^.

The number of atoms needed to represent each glass system in the AIMD simulations was determined from the mol% values. The total number of atoms was reduced by dividing by the highest common denominator (i.e., 5 for glass #1, 5 for simplified glasses #2, #3, #4, #5, and #6, and 2 for glass #4 when it was not simplified). This division resulted in preserving the ratio of the atoms while lowering the total number of atoms. To keep the total number of atoms in the system close to 200, the glass #1 composition and the simplified compositions were then multiplied by 2. The 200-atom system was chosen to obtain the most information with reasonable computation times using AIMD and with the expectation that the model would converge. There was no additional modification to the ‘not simplified’ case for glass #4. Modeled compositions are shown in Supplementary Table 1.

### Supplementary Information

Supplementary Information.

## Data Availability

All data generated or analyzed during this study are included in this published article (and its Supplementary Information files).
