# Supplementary material for: Antimicrobial Fe2O3-CuO-P2O5 glasses
Source: Sci Rep. 2023 Oct 14;13:17472. doi: 10.1038/s41598-023-44743-x (PMC10576775; doi:10.1038/s41598-023-44743-x)
Supplement: Supplementary file 1 — Supplementary Information. [file 41598_2023_44743_MOESM1_ESM.docx]

**Supplementary Information**

**Title: Antimicrobial Fe_2_O_3_-CuO-P_2_O_5_ glasses**

**Authors:** Alexandra L. Mitchell^1*^, SungHoon Lee^2^, David J. McEnroe^1^, Eric L. Null^1^, Daniel A. Sternquist^1^, Kathryn A. Hufziger^1^, Brian J. Rice^1^, Alex Scrimshire^3^, Paul A. Bingham^3^, Timothy M. Gross^1^

**Author Affiliations:** ^1^Corning Incorporated, 1 Riverfront Plaza, Corning, NY 14831, USA.

^2^Corning Technology Center Korea, Corning Precision Materials Co., Ltd., 212 Tangjeong-ro, Asan, Chungcheongnam-do 31454, Republic of Korea*.*

^3^Materials and Engineering Research Institute, Sheffield Hallam University, City Campus, Sheffield, S1 1WB, United Kingdom.

*****Corresponding author’s email: [mitchellal@corning.com](mailto:mitchellal@corning.com)

**Supplementary Table 1.** FeCuP glass compositions used for the AIMD simulations, taking oxidation state into account. The Fe content for glass #6 was lowered and P content raised compared to experimental values so that the composition could be distinguished from glass #5.

| **# Atoms** | **1** | **2** | | **3** | **4** | | **4** | **5** | | **6** |
| --- | --- | --- | --- | --- | --- | --- | --- | --- | --- | --- |
|  | **Not Simplified** | | **Simplified** | | | **Not Simplified** | | | **Simplified** | |
|  | **(No Fe)** | | **(All Fe as FeO)** | | | **(FeO & Fe_2_O_3_)** | | | **(All Fe as FeO)** | |
|  |  | |  | | |  | | |  | |
| **P** | 44 | 44 | | 44 | 44 | | 55 | 44 | | 48 |
| **Fe** | 0 | 2 | | 4 | 6 | | 9 | 4 | | 2 |
| **Cu** | 24 | 20 | | 22 | 20 | | 25 | 20 | | 20 |
| **O** | 128 | 128 | | 128 | 128 | | 163 | 128 | | 136 |
| **Sum** | 196 | 194 | | 198 | 198 | | 252 | 196 | | 206 |
|  |  |  | |  |  | |  |  | |  |
| ***O/P Ratio*** | *2.91* | *2.91* | | *2.91* | *2.91* | | *2.96* | *2.91* | | *2.83* |

**Supplementary Table 2.** FeCuP glass compositions (Series A = Glasses #1-#4 and Series B = Glasses #4-#6)

| **Analyzed composition (mol%)** | 1 | 2 | 3 | 4 | 5 | 6 |
| --- | --- | --- | --- | --- | --- | --- |
| **P_2_O_5_** | 46.4 | 46.1 | 45.3 | 44.7 | 47.5 | 49.4 |
| **Fe_2_O_3_** | 0.0 | 4.3 | 8.7 | 13.1 | 9.1 | 8.8 |
| **CuO** | 53.2 | 49.4 | 45.3 | 41.2 | 42.2 | 40.5 |
| **SiO_2_** | 0.5 | 0.3 | 0.7 | 1.0 | 1.2 | 1.3 |

**Supplementary Table 3.** Analyzed atom ratios in bulk glasses and in the Day 5 solution showing that about half the expected P was released during leaching. Data in 3a is normalized to Cu (as the largest component of the leachate) and in 3b is normalized to P (as the glass former).

| *Table 3a* |  | |  | |
| --- | --- | --- | --- | --- |
|  | *Initial* | | *Day 5 Solution* | |
| Glass # | **Fe/Cu** | **P/Cu** | **Fe/Cu** | **P/Cu** |
| **1** | 0.00 | 1.74 | 0.00 | 0.76 |
| **2** | 0.17 | 1.86 | 0.15 | 0.89 |
| **3** | 0.38 | 2.00 | 0.30 | 0.87 |
| **4** | 0.64 | 2.17 | 0.51 | 1.00 |
| **5** | 0.43 | 2.25 | 0.41 | 1.05 |
| **6** | 0.44 | 2.44 | 0.37 | 1.16 |

| *Table 3b* |  | |  | |
| --- | --- | --- | --- | --- |
|  | *Initial* | | *Day 5 Solution* | |
| **Glass #** | **Fe/P** | **Cu/P** | **Fe/P** | **Cu/P** |
| **1** | 0.00 | 0.57 | 0.00 | 1.32 |
| **2** | 0.09 | 0.54 | 0.17 | 1.13 |
| **3** | 0.19 | 0.50 | 0.35 | 1.15 |
| **4** | 0.29 | 0.46 | 0.51 | 1.00 |
| **5** | 0.19 | 0.44 | 0.39 | 0.96 |
| **6** | 0.18 | 0.41 | 0.32 | 0.87 |

**Supplementary Table 4.** The center shifts (CS) and quadrupole splitting (QS) obtained from room temperature Mössbauer spectra for Fe^2+^ and Fe^3+^ ions. Redox ratios were calculated assuming the room temperature recoil-free fraction ratio *f*(Fe^3+^) / *f*(Fe^2+^) = 1.3.

|  | Glass #2 | | Glass #3 | | Glass #4 | | Glass #5 | | Glass #6 | |
| --- | --- | --- | --- | --- | --- | --- | --- | --- | --- | --- |
| Center Shift (mm s^-1^) | 0.40 | 1.33 | 0.44 | 1.20 | 0.44 | 1.25 | 0.45 | 1.22 | 0.48 | 1.27 |
| Quadropole Splitting (mm s^-1^) | 0.65 | 2.27 | 0.62 | 2.52 | 0.82 | 2.28 | 0.57 | 2.33 | 0.65 | 2.25 |
| Fe^2+^/ΣFe (%) | 58 | 42 | 53 | 47 | 35 | 65 | 40 | 60 | 45 | 55 |
| Assignment | (III) | (II) | (III) | (II) | (III) | (II) | (III) | (II) | (III) | (II) |
| Reduced Χ^2^ | 0.621 | | 0.698 | | 0.783 | | 0.595 | | 0.594 | |

**Supplementary Table 5.** AIMD results corresponding to data shown in Figure 5.

| **% Bond Type** | **1** | **2** | **3** | **4** | **4** | **5** | **6** |
| --- | --- | --- | --- | --- | --- | --- | --- |
|  | **Not Simplified** | **Simplified** | | | **Not Simplified** | **Simplified** | |
|  | **(No Fe)** | **(All Fe as FeO)** | | | **(FeO & Fe_2_O_3_)** | **(All Fe as FeO)** | |
|  |  |  |  |  |  |  |  |
| **P–O–P** | 37.5 ± 0.0 | 38.6 ± 1.2 | 36.9 ± 0.7 | 36.1 ± 0.7 | 35.4 ± 1.6 | 37.5 ± 0.8 | 37.2 ± 0.7 |
| **P–O–Fe, Fe** | | 0.2 ± 0.3 | 0.2 ± 0.3 | 1.3 ± 0.8 | 1.6 ± 0.5 | 0.5 ± 0.4 | 0.2 ± 0.4 |
| **P–O–Fe** |  | 6.9 ± 2.8 | 13.6 ± 1.9 | 18.6 ± 1.9 | 18.8 ± 2.7 | 13.1 ± 2.5 | 6. 5± 1.4 |
| **Fe–O–Fe** |  | 0.5 ± 0.7 | 1.1 ± 0.4 | 1.3 ± 0.9 | 2.5 ± 0.0 | 0.6 ± 0.9 | 0.2 ± 0.4 |
| **Fe–O–x** |  | 0.8 ± 0.6 | 0.2 ± 0.3 | 0.2 ± 0.3 | 0.4 ± 0.5 | 0.9 ± 0.4 | 2.5 ± 0.4 |
| **P–O–x** | 62.5 ± 0.0 | 53.1 ± 0.6 | 48.1 ± 1.4 | 42.7 ± 0.9 | 41.3 ± 0.9 | 47.3 ± 1.4 | 53.5 ± 0.7 |
| **Sum** | 100 | 100 | 100 | 100 | 100 | 100 | 100 |

**
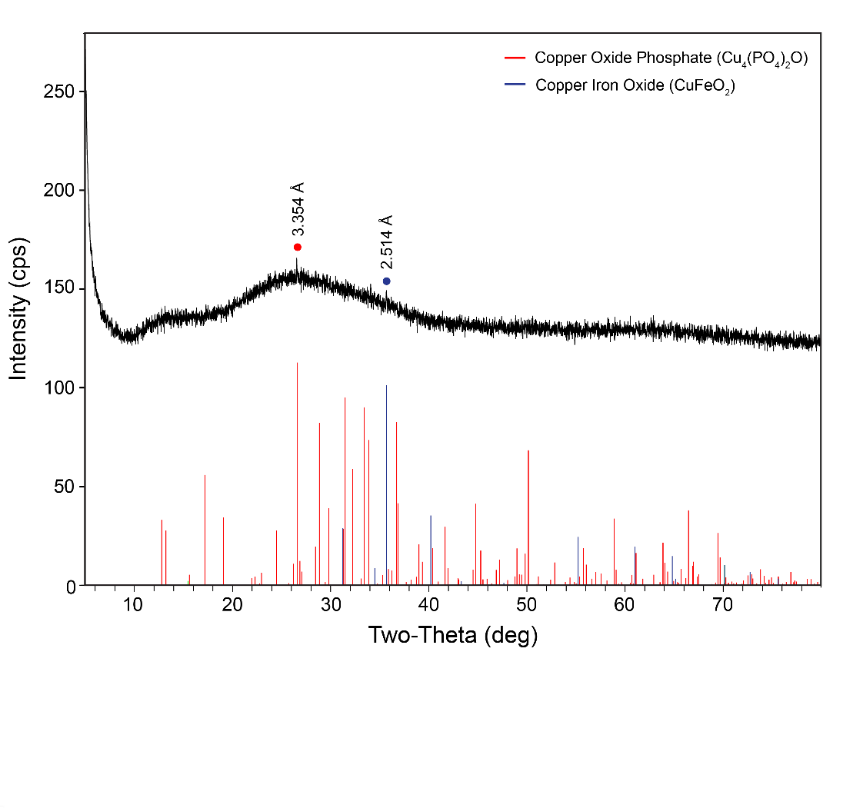
**

**Supplementary Figure 1.** XRD data for glass #4 showed low levels of crystallinity.

**Supplementary Figure 2.** Room temperature ^57^Fe Mössbauer spectra and fits for glasses **a)** #2, **b)** #3, and **c)** #4 (top to bottom), with CS relative to α-Fe.

**Supplementary Figure 3.** Average coordination number for oxygen, which links neighboring P- and Fe-polyhedra as a function of glass #. Values were determined by *ab-initio* molecular dynamics simulations for the analyzed compositions.

**Supplementary Figure 4.** The amount of leached a) Fe and b) P as a function of Fe_2_O_3_ content (mol%).
